# Complete Genome Sequence of the Polychlorinated Biphenyl Degrader *Rhodococcus* sp. WB1

**DOI:** 10.1128/genomeA.00996-16

**Published:** 2016-10-13

**Authors:** Yuxin Xu, Man Yu, Alin Shen

**Affiliations:** Environmental Resources and Soil Fertilizer Institute, Zhejiang Academy of Agricultural Sciences, Hangzhou, China

## Abstract

*Rhodococcus* sp. WB1 is a polychlorinated biphenyl degrader which was isolated from contaminated soil in Zhejiang, China. Here, we present the complete genome sequence. The analysis of this genome indicated that a biphenyl-degrading gene cluster and several xenobiotic metabolism pathways are harbored.

## GENOME ANNOUNCEMENT

Polychlorinated biphenyls (PCBs) are manufactured chemicals and were widely used as transformer fluids, hydraulic fluids, and other industrial products between the 1930s and 1980s because of their excellent physical and chemical properties (1, 2). PCBs are highly toxic pollutants that are harmful to the human immune, reproductive, nervous, and endocrine systems due to their difficultly for degradation and their bioaccumulation (3). PCBs can be degraded by microorganisms, and many degrading bacteria have been isolated since 1970, include Gram-negative bacteria and Gram-positive strains (4). *Rhodococcus* is a genus with the versatile catabolic ability to metabolize a large number of organic compounds (5, 6). Several *Rhodococcus* strains that can degrade PCBs have been isolated from various environments in past decades (5). *Rhodococcus* sp. WB1 was isolated from PCB-contaminated soil in Zhejiang, China. It can degrade numerous PCB congeners, from monochlorides to tetrachlorides.

The *Rhodococcus* sp. WB1 genome was sequenced by Shanghai Majorbio Bio-Pharm Technology Co., Ltd. (Shanghai, China) using the Illumina HiSeq and PacBio RS systems. An Illumina HiSeq paired-end library (500 bp) and a PacBio library (8 to 10 kb) were constructed. The raw sequences comprised 1.20 Gb, with an approximately 195-fold coverage of the genome. The data were assembled to contigs and finally to two scaffolds using SOAPdenovo version 2.04 (7) and Celera Assembler version 8.0 (8). The genome of WB1 has a circular chromosome and a plasmid, the bigger one is 5,924,027 bp long, and the plasmid is 224,533 bp, with average G+C contents of 70.50%. The genome of strain WB1 was analyzed and annotated. Fifty-four tRNAs were indicated by tRNAscan-SE version 1.3.1 (9), and 12 rRNAs were predicated by Barrnap version 0.4.2. The annotation of open reading frames was performed using Glimmer version 3.0 (10) and compared with the COG, NR, KEGG, String, GO, and Swiss-Prot databases by BLASTp (BLAST version 2.2.28). The big circle genome contains 3,072 predicated proteins and the small plasmid contains 31 proteins.

The WB1 genome contained a PCB degradation *bph* gene cluster (order: *bphB*, *bphA3A2A1*, *bphC*, *bphD*, and *bphA4*), which is unlike other PCB degraders (3, 11). In addition, the WB1 genome contained more than 10 xenobiotic metabolism pathways and 284 related genes, such as PCB degradation, nicotinate and nicotinamide metabolism, polycyclic aromatic hydrocarbon degradation, toluene and nitrotoluene degradation, and atrazine degradation. It suggested that WB1 is a potential metabolically versatile genus for persistent organic pollutants.

### Accession number(s).

This whole-genome shotgun project has been deposited in DDBJ/ENA/GenBank under the accession numbers CP015529 (chromosome) and CP015530 (plasmid). The versions described in this paper are the first versions, CP015529.1 and CP015530.1.
